# Pannexin 3 regulates proliferation and differentiation of odontoblasts via its hemichannel activities

**DOI:** 10.1371/journal.pone.0177557

**Published:** 2017-05-11

**Authors:** Tsutomu Iwamoto, Takashi Nakamura, Masaki Ishikawa, Keigo Yoshizaki, Asuna Sugimoto, Hiroko Ida-Yonemochi, Hayato Ohshima, Masahiro Saito, Yoshihiko Yamada, Satoshi Fukumoto

**Affiliations:** 1 Department of Pediatric Dentistry, Institute of Biomedical Sciences, Tokushima University Graduate School, Kuramoto-cho, Tokushima, Japan; 2 Division of Pediatric Dentistry, Tohoku University Graduate School of Dentistry, Sendai, Miyagi, Japan; 3 Division of Operative Dentistry, Tohoku University Graduate School of Dentistry, Sendai, Miyagi, Japan; 4 Laboratory of Cell and Developmental Biology, National Institute of Dental and Craniofacial Research, NIH, Bethesda, Maryland, United States of America; 5 Section of Orthodontics and Dentofacial Orthopedics, Kyushu University Faculty of Dental Science, Maidashi, Fukuoka, Japan; 6 Division of Anatomy and Cell Biology of the Hard Tissue, Niigata University Graduate School of Medical and Dental Sciences, Gakkocho-dori, Chuo-ku, Niigata, Japan; The University of Hong Kong, HONG KONG

## Abstract

Highly coordinated regulation of cell proliferation and differentiation contributes to the formation of functionally shaped and sized teeth; however, the mechanism underlying the switch from cell cycle exit to cell differentiation during odontogenesis is poorly understood. Recently, we identified pannexin 3 (Panx3) as a member of the pannexin gap junction protein family from tooth germs. The expression of Panx3 was predominately localized in preodontoblasts that arise from dental papilla cells and can differentiate into dentin-secreting odontoblasts. Panx3 also co-localized with p21, a cyclin-dependent kinase inhibitor protein, in preodontoblasts. *Panx3* was expressed in primary dental mesenchymal cells and in the mDP dental mesenchymal cell line. Both Panx3 and p21 were induced during the differentiation of mDP cells. Overexpression of *Panx3* in mDP cells reduced cell proliferation via up-regulation of p21, but not of p27, and promoted the Bone morphogenetic protein 2 (BMP2)-induced phosphorylation of Smad1/5/8 and the expression of dentin sialophosphoprotein (*Dspp*), a marker of differentiated odontoblasts. Furthermore, Panx3 released intracellular ATP into the extracellular space through its hemichannel and induced the phosphorylation of AMP-activated protein kinase (AMPK). 5-Aminoimidazole-4-carboxamide-ribonucleoside (AICAR), an activator of AMPK, reduced mDP cell proliferation and induced p21 expression. Conversely, knockdown of endogenous *Panx3* by siRNA inhibited AMPK phosphorylation, p21 expression, and the phosphorylation of Smad1/5/8 even in the presence of BMP2. Taken together, our results suggest that Panx3 modulates intracellular ATP levels, resulting in the inhibition of odontoblast proliferation through the AMPK/p21 signaling pathway and promotion of cell differentiation by the BMP/Smad signaling pathway.

## Introduction

Cellular communication enabling cross-talk with the extracellular environment, including neighboring cells and the extracellular matrix, is crucial for coordinating and synchronizing the pattern and rate of cell division during organ development. Gap junctions, consisting of the connexin (Cx) and pannexin (Panx) protein families, play a crucial role in cellular communication by mediating the transfer of ions (K^+^ and Ca^2+^), second messengers (cAMP, ATP, and inositol 1,4,5-trisphosphate), and other metabolites (glucose) as specialized transmembrane channels [1–3].

Connexins (Cxs) are implicated in a variety of cellular functions including embryonic development and cellular differentiation as well as proliferation and migration [4]. Cx43, one of the most prevalent connexin proteins, is widely expressed in neural crest cell lineages that contribute to a diverse array of structures in the embryo including the peripheral nervous system, cranial mesenchyme, and cardiac morphogenesis [5]. Mutations in the Cx43 gene (GJA1) are associated with oculodentodigital dysplasia (ODDD), an autosomal dominant syndrome characterized by craniofacial and limb dysmorphology, spastic paraplegia, and neurodegeneration [6]. Mutations in the either Cx26 or Cx30 cause a variety of nonsyndromic congenital deafness and hyperkeratosis syndromes [7, 8]. In addition, Cx43 knockout mice exhibit neonatal lethality due to conotruncal heart defects similar to ODDD [6, 9] and Cx36 knockout mice show impaired hippocampal gamma oscillations [10]. Thus, each connexin has both specific and redundant functions in development and cell behavior.

Pannexins, homologous to the invertebrate gap junction innexin proteins, were recently identified as a second vertebrate gap junction protein family [3]. Although connexins and pannexins share similar morphological structures, they have no sequence homology [11]. Pannexins are comprised of three members, pannexin 1, 2, and 3 (Panx1, 2, 3). Panx1 is ubiquitously expressed, with the highest levels of expression observed in the developing and mature central nervous system [11]. Panx1 functions as an ATP hemichannel that responds to intracellular Ca^2+^ levels and lowered O_2_ tension [12, 13]. Panx2 is highly expressed in the central nervous system [11], while Panx3 is strongly expressed in developing cartilage and bone [14–16]. The Panx3 ATP hemichannel functions to switch the cell fate of chondrocytes and osteoblasts from proliferation to differentiation by regulating intracellular ATP/cAMP levels [14–16]. Unlike connexins, Panx3 acts as an endoplasmic reticulum (ER) Ca^2+^ channel that modulates intracellular Ca^2+^ signaling during osteoblast differentiation [15]. In addition, Panx3 is induced by Bone morphogenetic protein 2 (BMP2) and a target gene of Runt-related transcription factor 2 (Runx2), which is a key transcription factor for bone formation [17]. Panx3 knockout mice evidently demonstrated that Panx3 is essential for normal skeletal development [18, 19]. Thus, Panx3 plays a crucial role in hard tissue development.

The tooth is a unique hard tissue consisting of enamel and dentin. Tooth development commences with the invagination of dental epithelial cells into the underlying cranial neural crest-derived ectomesenchymal cell layer. Subsequently, the ectomesenchymal cells condense to form the dental papilla. The outer cells of the dental papilla that come in contact with the basement membrane underlying the dental epithelial cells differentiate into preodontoblasts, creating a contiguous monolayer in the peripheral cell region of the dental papilla. The basement membrane is then degraded and the preodontoblasts differentiate into dentin-matrix secreting odontoblasts, which are responsible for the formation and maintenance of the dentin. The intercellular communication of odontoblasts mediated via gap junctions is thought to play an important role in the ordered tubular and layered structure of the dentin. In fact, the existence of gap junctions between odontoblasts has been demonstrated by freeze fracture studies [20] and transjunctional flux of fluorescent tracers [21]. Weak expression of Cx32 was observed in differentiating odontoblasts [22] and the expression of Cx43 was increased in differentiated odontoblasts [22, 23]. These observations suggest that gap junctions between odontoblasts coordinate cellular activity. However, the expression and physiological function of Panx3 in tooth development have not been clearly elucidated.

In this study, we demonstrated that Panx3 is expressed in preodontoblasts and regulates preodontoblast proliferation and differentiation. The Panx3 ATP hemichannel regulates the AMP-activated protein kinase (AMPK) signaling pathway and induces cell cycle exit through the upregulation of p21 expression. Thus, Panx3 plays important roles in the regulation of the transition stage from proliferation to differentiation in odontoblasts.

## Materials and methods

### RT-PCR and northern hybridization

Total RNA was extracted from newborn mouse tissues or cells using the Trizol reagent kit (Invitrogen). Following DNase I (Sigma) treatment, 1 μg of total RNA was used to generate cDNA, which was used as template for PCR with the following gene-specific primers: *Panx3*, 5′-GCCCCTGGATAAGATGGTCAAG-3′ and 5′-GCGGATGGAACGGTTGTAAGA-3′; *Ambn*, 5′-GCGTTTCCAAGAGCCCTGATAAC-3′ and 5′-AAGAAGCAGTGTCACATTTCCTGG-3′; *Panx1*, 5′-TTTGGACCTAAGAGACGGACCTG-3′ and 5′-CGGGAATCAGCAGAGCATACAC-3’; *Dspp*, 5′-CTCAGAGAGAATCTGGGTGTACCACC-3′ and 5′-CACAGTGGTACATGGAGAGCTC-3′; *Cx43*, 5′-CAAGGAGTTCCACCACTTTGGC-3′ and 5′-GAAAATGAAGAGCACCGACAGC-3′; *Gapdh*, 5′-ACCACAGTCCATGCCATCAC-3′ and 5′-TCCACCACCCTGTTGCTGTA-3′. PCR reactions were carried out using a Gene Amp PCR system 9700 (Applied Biosystems) with the following conditions: 94°C for 3 min and 25–33 cycles of 94°C for 30 s, 60°C for 30 s, and 72°C for 1 min. PCR products were separated by electrophoresis on 1.5% agarose gels, which were stained with ethidium bromide and photographed. Real-time PCR (RT-qPCR) amplification was performed using primers with SYBR Green PCR Master Mix and a StepOne^™^ real-time PCR System (Applied Biosystems, Foster City, CA, USA). For Northern blotting, 20 μg of total RNA was separated by electrophoresis and transferred to a Nytran membrane (Schleicher & Schuell), as previously described [24]. cDNA was labeled with [α-^32^P] dCTP using Ready-To-Go DNA labeling beads (Amersham Biosciences). The membranes were hybridized with labeled probes at 68°C in QuikHyb (Stratagene), washed first at 65°C in 1× SSC with 0.1% SDS and then at 65°C in 0.1× SSC with 0.1% SDS and then exposed to autoradiography film (Kodak).

### In situ hybridization

Digoxigenin-11-UTP-labeled single-stranded antisense RNA probes for *Panx3* and *Dspp* were prepared using the DIG RNA labeling kit (Roche Applied Science) according to the manufacturer’s instructions. *In situ* hybridization of the tissue sections was performed according to the protocol provided with the Link-Label ISH Core Kit II (BioGenex). Frozen tissue sections were obtained from postnatal day 1 (P1) mouse embryo heads containing molars and incisors and placed on RNase-free glass slides. The frozen sections were dried for 10 min at room temperature and incubated at 37°C for 30 min then treated with 10 μg/mL proteinase K at 37°C for 30 min. Hybridization was performed at 37°C for 16 h. Washes were carried out with 2 × SSC at 50°C for 15 min and 2 × SSC containing 50% formamide at 37°C for 15 min. The sections were then treated with 10 μg/mL RNase A in 10 mM Tris-HCl (pH 7.6), 500 mM NaCl, and 1 mM EDTA at 37°C for 15 min and then washed. The sections were treated with 2.4 mg/mL Levamisole (Sigma) to inactivate endogenous alkaline phosphatase.

### Immunohistochemistry

Frozen tissue sections were fixed with acetone at -20°C for 2 min or 4% paraformaldehyde at room temperature for 5 min and then washed three times in PBS for 5 min. Immunohistochemistry was performed on sections incubated with Universal Blocking Reagent (BioGenex) in 1 × PBS/0.3% Triton X-100 for 7 min at room temperature prior to incubation with the primary antibody. Antibodies against p21 (C-19; Santa Cruz), Lamb1 (Abcam), and Cx43 (US Biological) were used. A rabbit polyclonal antibody against the Panx3 peptide (residues 90–107) was raised and purified using a peptide affinity column. Primary antibodies were detected by Cy-3- or Cy-5-conjugated secondary antibodies (Jackson ImmunoResearch Laboratories). Nuclear staining was performed with DAPI (Invitrogen). A fluorescent microscope (Axiovert 200; Carl Zeiss MicroImaging, Inc., Biozero-8000; Keyence, Japan) was used for immunofluorescence image analysis. Images were prepared using AxioVision and Photoshop (Adobe Systems, Inc.).

### Electron microscopy

One-day-old ICR mice were perfused with 4% paraformaldehyde in a 0.1M phosphate buffer (pH7.4) under deep anesthesia by an intraperitoneal injection of chloral hydrate (500 mg/kg). Following decalcification with a 4.13% EDTA-2Na solution, frozen sections were cut sagittally at 80 μm. For immunohistochemistry at the electron-microscopic level, the sections were processed for the Envision+/horseradish peroxidase system (Dako Japan) using anti-Panx3 antibody diluted to 1:100. For final visualization of the sections, 0.05M Tris-HCl buffer (pH 7.6) containing 0.04% 3–3′-diaminobenzidine tetrahydrochloride and 0.0002% H_2_O_2_ was used. For transmission electron microscopy, the immunostained tissues were postfixed in 1% OsO_4_ reduced with 1.5% potassium ferrocyanide, dehydrated through ethanol series, and embedded in Epon 812 (Taab, Berkshire, UK). Semithin sections were cut at 1 μm and stained with 0.03% methylene blue. Ultrathin sections (70 nm in thickness) were double-stained with uranyl acetate and lead citrate and examined with an H-7650 transmission electron microscope (Hitachi High-Technologies Corp., Tokyo, Japan).

### Cell culturing

For primary cell cultures, first molars were dissected from P3 mice. To separate the dental epithelium from mesenchymal tissues, the samples were incubated for 10 min in 0.1% collagenase, 0.05% trypsin, and 0.5 mM EDTA and then transferred to keratinocyte-SFM supplemented with EGF and pituitary gland extract (PGE), according to the manufacturer's instructions. The tissues were then micro-surgically separated and then treated with 0.1% collagenase, 0.05% trypsin, and 0.5 mM EDTA for 15 min at 37°C. Dental epithelium specimens were plated in keratinocyte-SFM with PGE and either 2% FBS or EGF with the addition of 100 units/mL of penicillin and streptomycin and incubated at 37°C in a humidified atmosphere containing 5% CO_2_. Dental mesenchymal cells were plated on DMEM with 10% FBS as previously described [25]. A dental mesenchymal cell line (mDP) and a single cell clone cell derived from human deciduous tooth pulp cells (SDP11) were cultured in DMEM/F-12 with 10% FBS. Cells were maintained at 30–60% confluency and the medium was replaced every other day. For the differentiation of the cells, 200 ng/mL recombinant human Bone morphogenetic protein 2 (rhBMP2, Wako) was added to the culture medium. Panx3 peptides (HHTQDKAGQYKVK SLWPH from mouse Panx3 and HHKQDGPGPGQDKMKSLWPH from human Panx3) were used for inhibition experiments. A peptide with scrambled sequences (WHTKYQVGLDPQHKASHK for mouse and GMHWPHDPGDKLQKQKSH for human) of the Panx3 peptide were used as a control [14]. All animal experiments were approved by the ethics committee of Kyushu University Animal Experiment Center (protocol no. A19-039-0).

### Plasmid construction and transfection

The coding sequence of mouse *Panx3* cDNA was subcloned into the pEF1/V5-His vector (pEF1/Panx3) (Invitrogen). An empty pEF1vector was used as a control. For stable transfection, mDP cells were transfected with Lipofectamine 2000 (Invitrogen) according to the manufacturer's protocol. Selection was initiated 24 h post transfection, using G418 (Invitrogen) at a concentration of 600 μg/mL. Genetic recombination experiments were approved by the Research Center for Genetic Information, Kyushu University (19–105) and Institute for Genome Research, Tokushima University (25–39). All experiments were performed in accordance with the approved guidelines.

### Cell proliferation and bromodeoxyuridine (BrdU) incorporation

Cells were plated at 1 × 10^4^ cells/mL/well in 12-well plates and grown for 72 h. Cell numbers were determined using a trypan blue dye exclusion method. For the BrdU incorporation assay, cells were incubated at the same cell density described above for 48 h. BrdU (Sigma) was added to the plates (10 μM) for 30 min; then, the cells were fixed with cold methanol for 10 min, rehydrated in PBS, and incubated for 30 min in 1.5 M HCl. After three washes with PBS, the plates were incubated with a 1:50 dilution of fluorescein isothiocyanate-conjugated anti-BrdU antibody (Roche Applied Science) for 30 min at room temperature. Finally, the cells were washed three times with PBS and incubated with 10 μg/mL propidium iodide (Sigma) in PBS for 30 min at room temperature. BrdU-positive cells were examined under a microscope (Biozero-8000; Keyence, Japan).

### Western blotting

Cells were washed three times with PBS containing 1 mM sodium vanadate (Na_3_VO_4_), then solubilized in 100 μL lysis buffer (10 mM Tris-HCl [pH 7.4], 150 mM NaCl, 10 mM MgCl_2_, 0.5% Nonidet P-40, 1 mM PMSF, and 20 units/mL aprotinin). Lysed cells were centrifuged at 12,000 rpm for 5 min and the protein concentration of each sample was measured using the Micro-BCA Assay Reagent (Pierce Chemical Co.). The samples were denatured in SDS sample buffers and loaded on a 12% SDS-polyacrylamide gel. Ten micrograms of protein lysate were loaded in each lane. Subsequent to SDS-PAGE, the proteins were transferred onto a PVDF membrane and immunoblotted with antibodies to Panx3, p21 and p27 (Santa Cruz), β-actin (Abcam), and P-Smad1/5/8, Smad5, phospho-AMPKα, AMPKα, and retinoblastoma (Rb) (Cell Signaling) and then visualized using an ECL kit (Amersham Pharmacia Biotech). For the Smad and MAPK experiments, the cells were pretreated with siRNA as described below.

### siRNA experiments

Mouse Panx3 (NM_172454) siGenome On-Target plus (LQ-054624-01-0010; Dharmacon) was used to silence Panx3. ON-TARGET plus siCONTROL nontargeting pool siRNA (D-001810–10; Dharmacon) was used as a control. mDP cells were transfected with 100 nM siRNA duplex in regular serum-free culture medium using the Oligofectamine reagent (Invitrogen) according to the manufacturer's protocol.

### ATP flux

ATP flux was determined by luminometry. mDP cells were depolarized by incubation in KGlu solution (140 mM potassium gluconate, 10 mM KCl, and 5.0 mM TES, pH 7.5) for 10 min to open the pannexin channels. The supernatant was collected and assayed with luciferase/luciferin (Promega). For inhibition experiments, cells were treated with the Panx3-peptide or scramble peptide for 30 min prior to incubation in KGlu solution.

## Results

### Panx3 expression in mouse tissues

**Fig 1:**
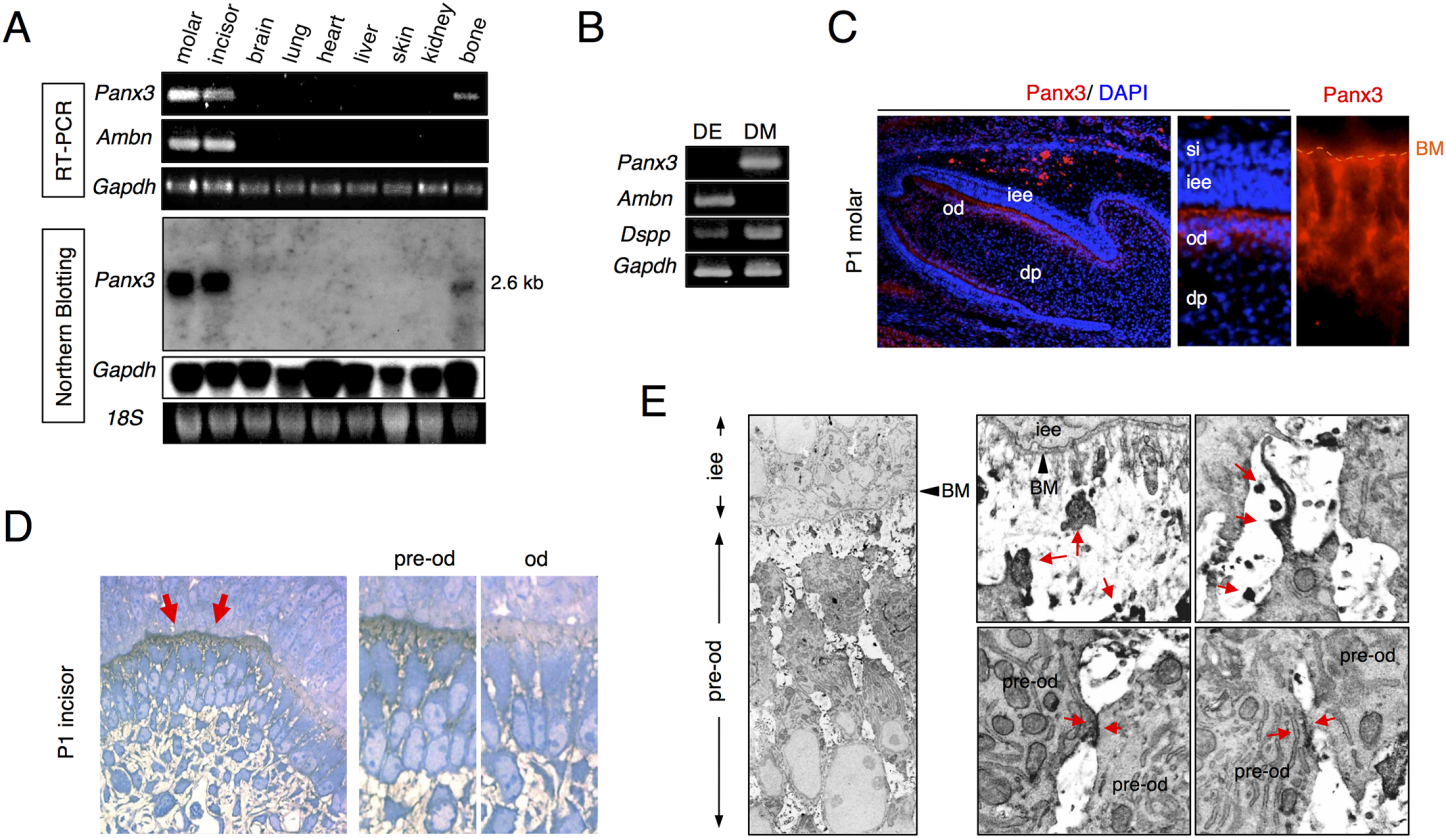
Expression of Panx3 in tooth germ. (A) RT-PCR analysis (upper three panels) and northern blotting analysis (lower three panels) using RNA from postnatal day 1 (P1) mouse tissues (molar, incisor, brain, lung, heart, liver, skin, kidney, and bone). (B) RT-PCR analysis using the dental epithelium (DE) and mesenchyme (DM), dissected from P1 mouse tooth germ. (C) Immnostaining with anti-Panx3 antibody (red) and DAPI nuclear staining (blue). (D) Light microscopy images of semi-thin sections stained with methylene blue for immunoelectron microscopy. Panx3 is present in the preodontoblasts (pre-od) but not in the odontoblasts (od). (E) Immunoelectron microscopy images of the Panx3 protein in preodontoblasts from P1 incisors showing labeling in the preodontoblasts (upper panels) and at the cell-cell contact sites (lower panels). dp; dental papilla, iee; inner enamel epithelium, si; stratum intermedium, BM; basement membrane.

We originally identified pannexin 3 (Panx3) from E19.5 mouse molar cDNA microarrays as preferentially expressed in teeth [24, 26]. To analyze the expression of *Panx3* mRNA in mouse tissues, we first performed RT-PCR and Northern blot analyses using RNA isolated from tissues of newborn mice. RT-PCR showed that *Panx3* was strongly expressed in molars and incisors (Fig 1A). *Panx3* expression was also detected in long bones but not in other tissues such as the brain, lung, heart, liver, skin and kidney. Ameloblastin, an enamel matrix marker was expressed only in molars and incisors. Northern blot analysis showed that *Panx3* was expressed as a single 2.6 kb mRNA band in molars, incisors, and long bones but not in other tissues (Fig 1A). Thus, the expression of *Panx3* mRNA is strong in hard tissues.

To analyze the cellular localization of *Panx3* in teeth, *Panx3* mRNA expression was first examined by RT-PCR using RNA from dental epithelium cells and mesenchyme cells prepared from postnatal day 1 (P1) molars. *Panx3* was expressed in the dental mesenchyme but not in the dental epithelium (Fig 1B). Next, we performed immunostaining (Fig 1C and 1D) and immunohistochemistry with an electron microscopic (Fig 1E) using a Panx3 specific antibody. Immunostaining revealed that Panx3 was strongly expressed in preodontoblasts but not in ameloblasts (Fig 1C and 1D). Panx3 was localized to cell-cell contact regions and cell processes facing the basement membrane (Fig 1C–1E)). These expression patterns indicate that Panx3 may function as a gap junction and a hemichannel.

### Panx3 expression in differentiating odontoblasts

**Fig 2:**
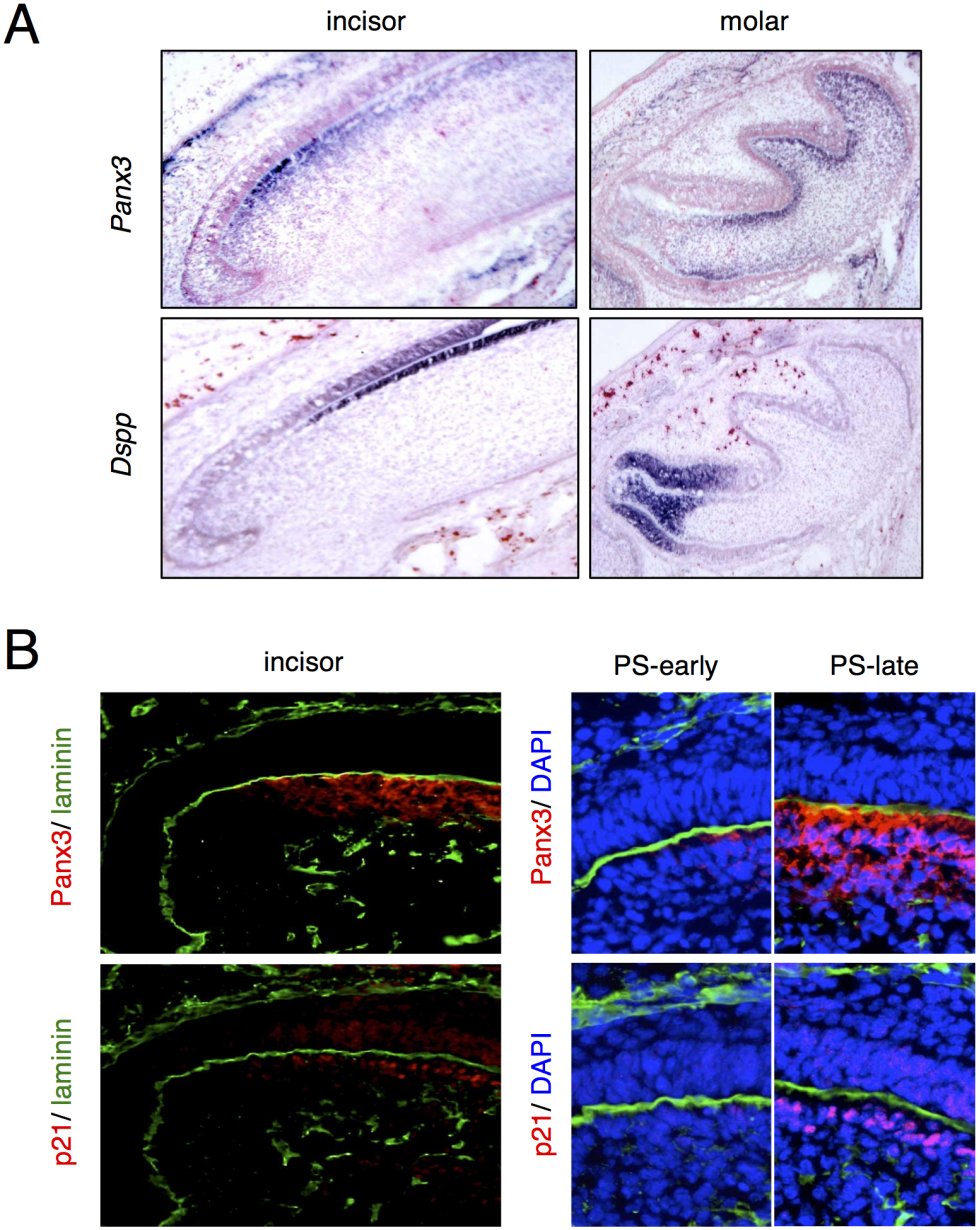
Expression of *Panx3* mRNA and protein in tooth germ. (A) *In situ* hybridization with *Panx3* and *Dspp* probes in postnatal day 1 (P1) incisor or molar semi-serial sections. (B) immunostaining with anti-Panx3 or anti-p21 antibodies (red) or laminin (green) in P1 incisor semi-serial sections. Blue signifies DAPI nuclear staining. PS; presecretory stage.

In order to further identify Panx3-expressing cells, we compared the mRNA expression pattern of *Panx3* and dentin sialophosphoprotein (*Dspp*), a maker of terminal differentiated odontoblasts and ameloblasts in incisors and molars (Fig 2A). The expression pattern of *Panx3* mRNA was different from that of *Dspp* mRNA, indicating that *Panx3* mRNA is expressed in preodontoblasts but not in differentiated mature odontoblasts (Fig 2A).

Preodontoblasts are characterized by proliferation to expand cell population and eventually stop proliferation for differentiation. Since p21 is considered to be important for cell growth arrest at the G1/S checkpoint in odontoblasts and ameloblasts [27], we compared the expression of Panx3 and p21 by immunostaining in incisor sections of P1 mice. In the incisor sections, we can observe gradual differentiation stages of odontoblasts. p21 expression was observed in the restricted area of odontoblasts and ameloblasts (Fig 2B). Panx3, but not p21, was expressed at the early presecretory stage of preodontoblasts, and both Panx3 and p21 expression was observed in the late presecretory stage of preodontoblasts (Fig 2B). These observations indicate that Panx3 expression slightly precedes the p21 expression in preodontoblasts, suggesting that Panx3 may regulate p21 expression.

### p21 expression in differentiating odontoblasts

**Fig 3:**
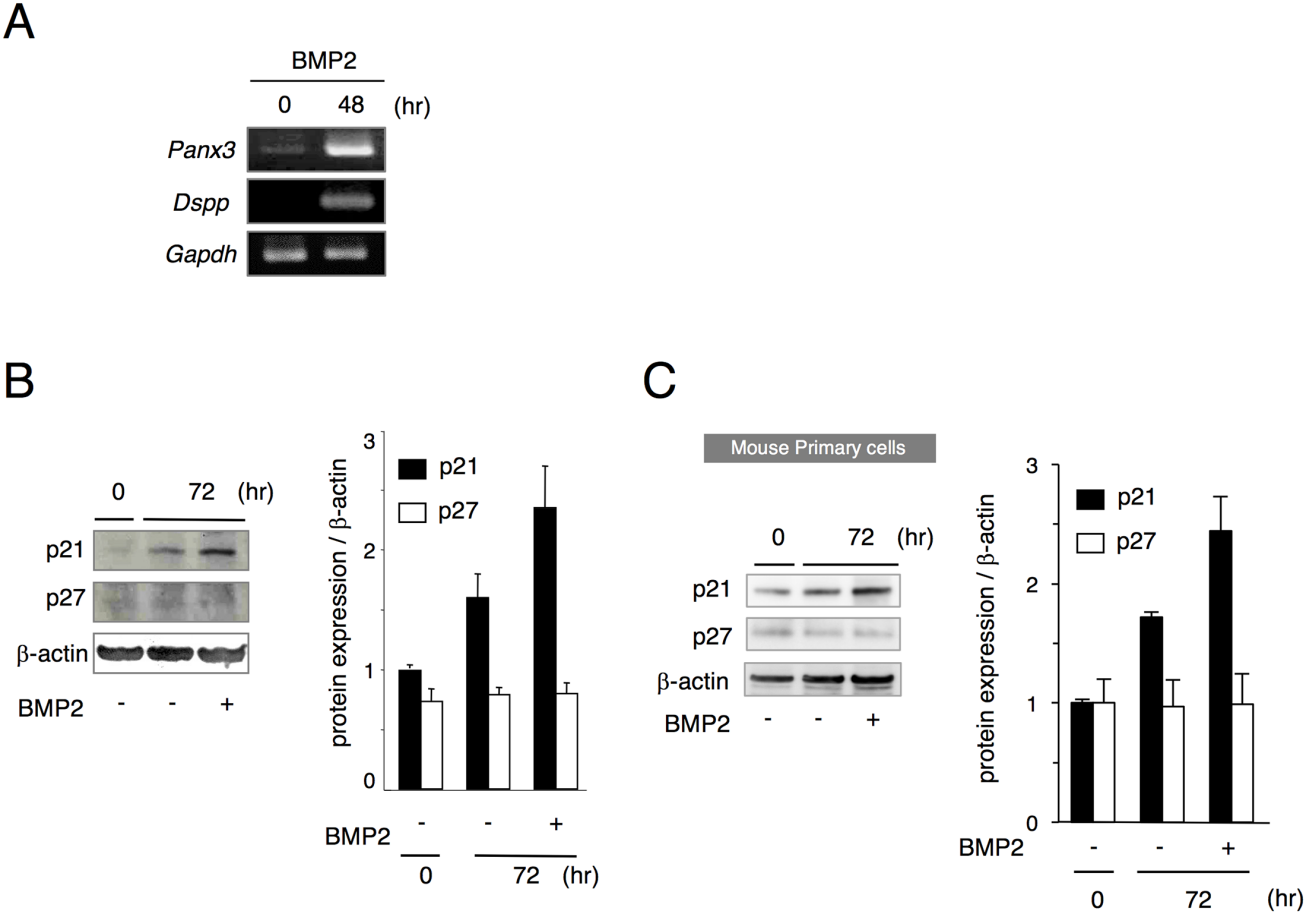
Expression of Panx3 and p21 in differentiating mDP cells and mouse primary dental papilla cells. (A) mRNA expression in differentiating mDP cells. mDP cells were cultured with 200 ng/mL BMP2. Total RNA was extracted from the cells at the indicated time points following BMP2 treatment and analyzed by RT-PCR. *Gapdh*, glyceraldehyde-3-phosphate dehydrogenase, was used as a control. p21 expression in differentiating mDP cells (B) and mouse primary dental papilla cells (C). Both mDP cells and mouse primary dental papilla cells were cultured with or without 200 ng/mL BMP2 for 72 h and cell extracts were analyzed by western blotting using anti-p21 and anti-p27 antibodies. β-actin was used as a control. Data were pooled from three independent experiments with error bars designating standard deviation of the mean.

To assess the role of Panx3 in odontoblast development, we used the mDP dental pulp cell line [28]. mDP cells can differentiate into *Dspp*-expressing cells in the presence of Bone morphogenetic protein 2 (BMP2). RT-PCR analysis showed that *Panx3* mRNA was weakly expressed in undifferentiated mDP cells and that the expression of *Panx3* and *Dspp* mRNA was induced in differentiating mDP cells (Fig 3A). Western blot analysis also demonstrated that the Panx3 protein was strongly induced during mDP differentiation (S1 Fig). The Panx3 protein size was approximated as 45-kDa by SDS-PAGE, corresponding to its predicted molecular weight. The Panx3 protein was not detected in undifferentiated mDP cells. These results indicate that Panx3 was induced during the differentiation of mDP cells.

p21 was co-expressed in Panx3-expressing preodontoblasts along with the basement membrane. We next examined whether p21 expression was also induced in mDP cells and mouse primary dental papilla cells undergoing BMP2-induced differentiation. Western blot analysis demonstrated that p21, but not p27, was induced by BMP2 treatment in mDP cells (Fig 3B) and mouse primary papilla cells (Fig 3C). These results demonstrate that p21 expression correlates with odontoblast differentiation.

### Inhibition of cell proliferation by Panx3

**Fig 4:**
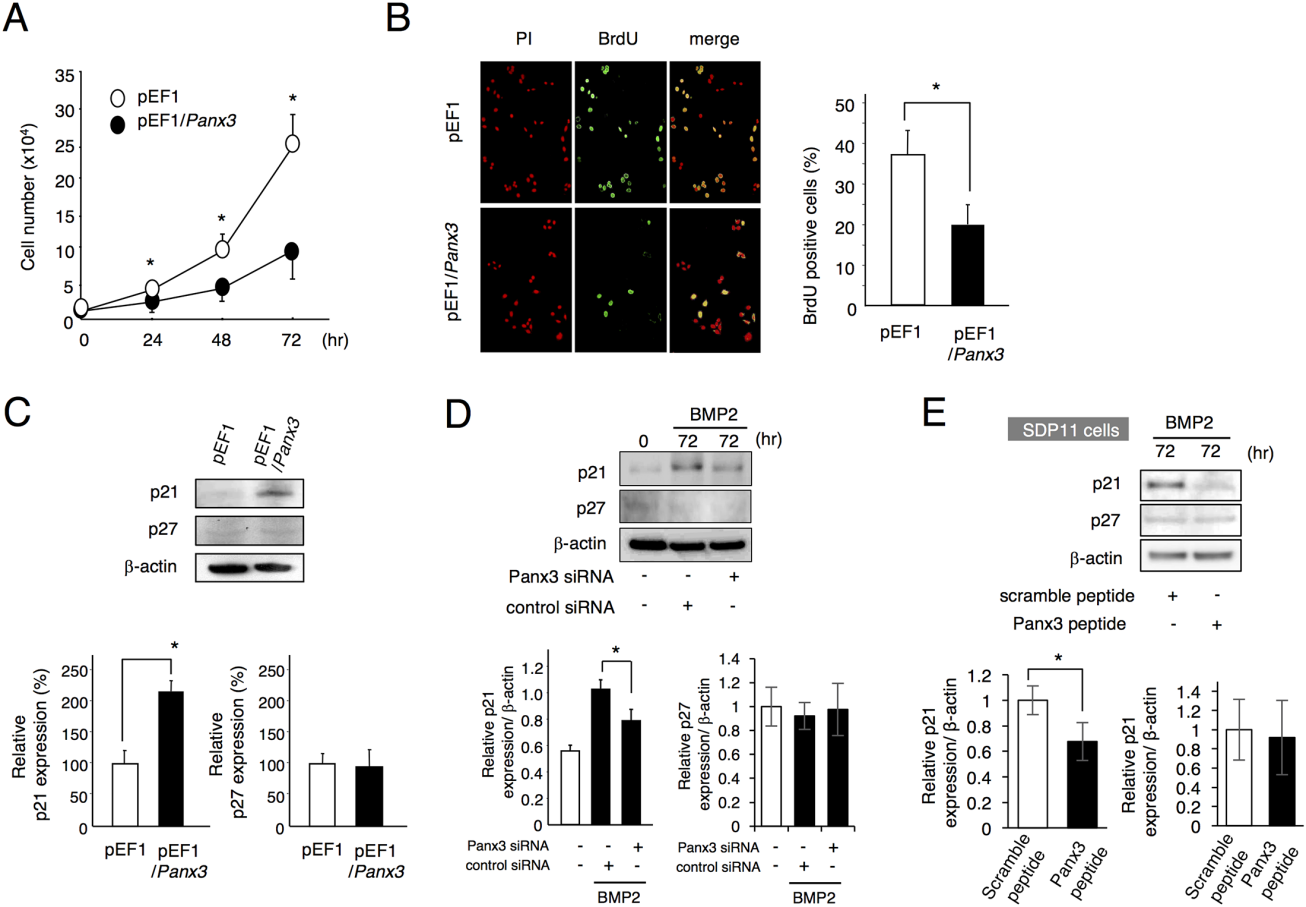
Inhibition of cell proliferation by Panx3. (A) mDP cells were stably transfected with the empty control vector (pEF1) or with the Panx3 expression vector (pEF1/*Panx3*). The cells were cultured for 72 h and cell numbers were counted. The number of *Panx3*-transfected cells was reduced compared to the control cells. (B) Cells were cultured for 48 h and BrdU incorporation after 30 min was analyzed using a fluorescence microscope. (C) Cell extracts were analyzed by western blotting using anti-p21 and anti-p27 antibodies. β-actin was used as a control. (D) mDP cells transfected with either control siRNA or *Panx3* siRNA, were cultured with 200 ng/ml BMP2 for 72 h. Western blotting was performed for p21 and p27 expressions. (E) SDP11 cells were cultured with 200 ng/mL BMP2 and 0.5 μg/ml Panx3 inhibitory peptide or control peptide for 72 h. Western blotting was performed for p21 and p27 expressions. All experiments were repeated at least three times with similar results. Data were pooled from three independent experiments with error bars designating standard deviation of the mean. Statistical analysis was performed using analysis of variance (*P < 0.01).

To analyze the function of Panx3 in odontoblast proliferation and differentiation, we established mDP cells stably expressing *Panx3* (pEF1/*Panx3*). We first examined whether Panx3 affects cell proliferation. *Panx3*-transfected mDP cells dramatically inhibited cell numbers in a three-day culture compared with control cells (Fig 4A). In addition, we analyzed the mDP cell proliferation using BrdU incorporation. The number of BrdU-positive cells decreased in *Panx3*-transfected mDP cells compared to control vector-transfected cells (Fig 4B). These results indicate that overexpression of *Panx3* inhibits mDP cell proliferation, suggesting that Panx3 inhibits dental mesenchymal cell proliferation *in vivo*. Since p21 is co-expressed in Panx3-expressing preodontoblasts, we next tested whether Panx3 affects p21 expression. Western blot analysis demonstrated that p21, but not p27, was significantly induced in *Panx3* overexpressing mDP cells (Fig 4C). To analyze the function of endogenous Panx3, we knocked down *Panx3* expression using *Panx3* siRNA transfection into mDP cells. BMP2-induced Panx3 expression was substantially reduced at the protein level compared to control siRNA transfected cells (S2 Fig). The expression of p21, but not of p27, was significantly reduced by the knockdown of endogenous *Panx3* (Fig 4D). Further, we have used single clone cell derived from deciduous tooth pulp cells, SDP11 cells [29]. SDP11 cells were cultured with BMP2 and Panx3 inhibitory peptide. The expression of p21, but not p27, was also significantly reduced by Panx3 inhibitory peptide (Fig 4E). These results indicate that the upregulation of endogenous Panx3 expression is required for p21 expression in BMP2-treated mDP cells and SDP11 cells, suggesting that the p21 expression correlates with the anti-proliferation activity of Panx3.

### Panx3 is necessary for Dspp expression

**Fig 5:**
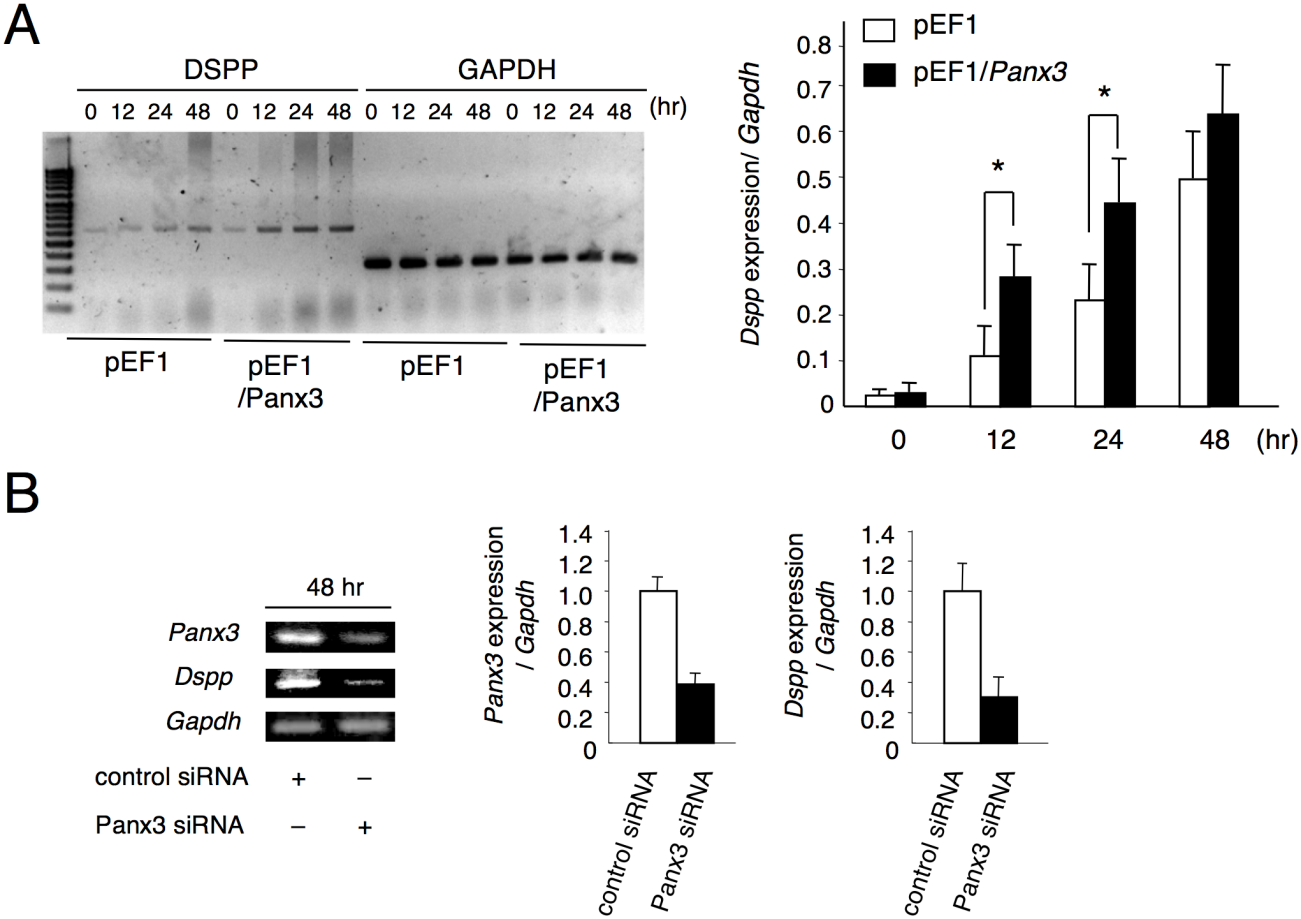
Increase in BMP2-mediated *Dspp* induction in mDP cells due to Panx3. (A) mDP cells stably transfected with either control pEF1 or pEF1/*Panx3* were cultured with 200 ng/mL of BMP2 for the indicated time. Total RNA was then extracted and analyzed by RT-PCR. (B) mDP cells transfected with either control siRNA or *Panx3* siRNA were cultured with the addition of 200 ng/mL BMP2 for 48 h. RT-PCR was performed to examine *Panx3* and *Dspp* expression. All experiments were repeated at least five times with similar results. Data were pooled from three independent experiments with error bars designating standard deviation of the mean. Statistical analysis was performed using analysis of variance (*P < 0.01).

We next examined whether overexpression of Panx3 affects mDP cell differentiation. *Panx3*-transfected mDP cells and control vector-transfected cells were cultured with BMP2. *Dspp* expression was strongly enhanced in *Panx3* overexpressing mDP cells following 12 and 24 h of culturing. In contrast, the expression of *Dspp* in control mDP cells gradually increased with 48 h of culturing (Fig 5A). Next, we analyzed whether knockdown of endogenous *Panx3* affects mDP cell differentiation. Suppression of endogenous Panx3 by *Panx3* siRNA inhibited BMP2-induced *Dspp* expression by approximately 70% (Fig 5B). These results indicate that Panx3 promotes mDP cell differentiation and is necessary for odontoblast differentiation.

### Panx3 affects the phosphorylation of Smad1/5/8

**Fig 6:**
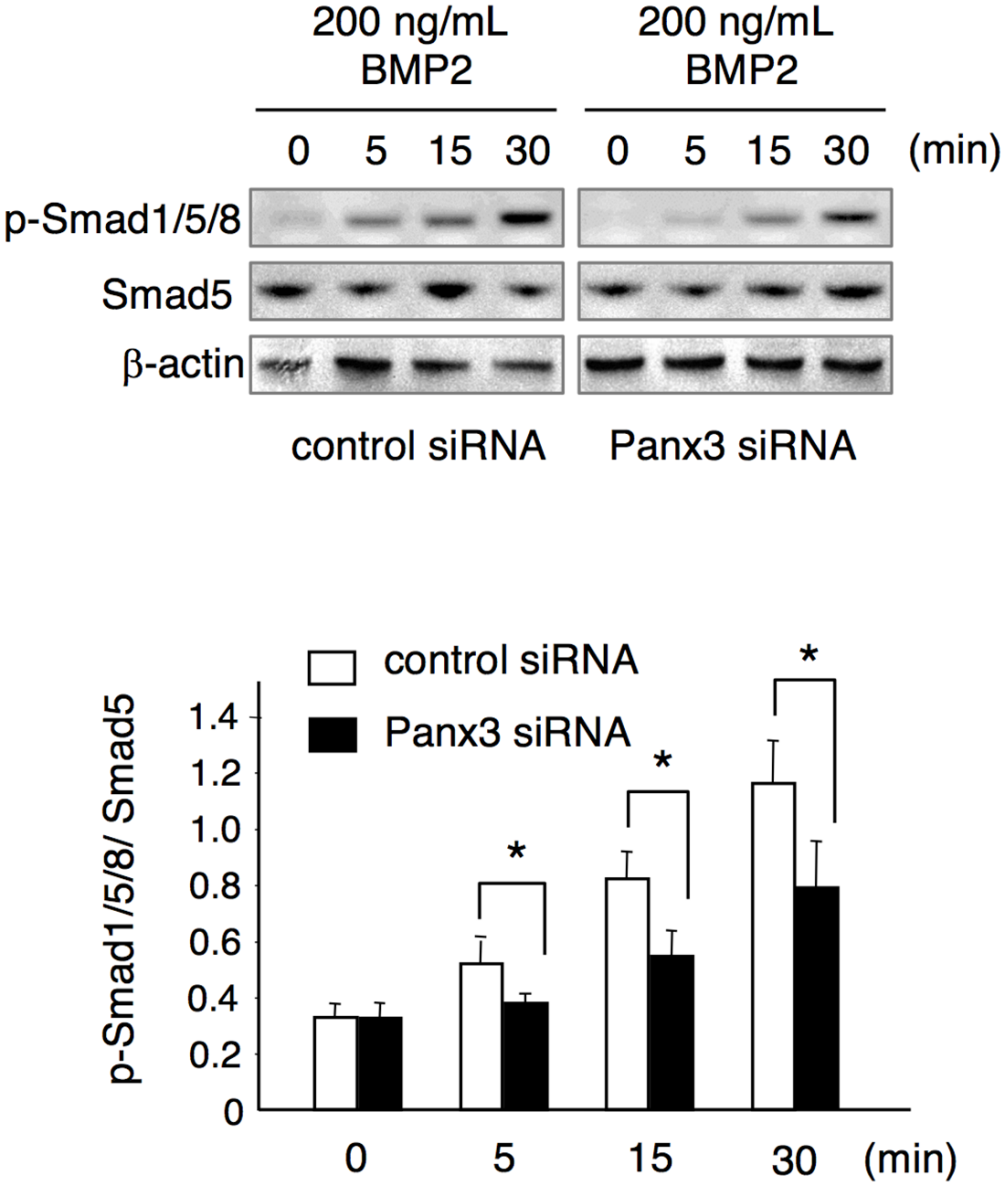
Decrease in BMP2-induced Smad1/5/8 phosphorylation due to Panx3 siRNA. Following transfection with either control siRNA or *Panx3* siRNA for 48 h, cells were treated with 200 ng/mL BMP2 for the time indicated. Protein extracts were analyzed by western blotting using anti-phospho-Smad1/5/8, anti-Smad5, and anti-β-actin antibodies. *Image J* 1.33u was used to quantify the protein bands. Data were pooled from three independent experiments with error bars designating standard deviation of the mean. Statistical analysis was performed using analysis of variance (*P < 0.01).

Since Panx3 is involved in *Dspp* expression and the phosphorylation of Smad1/5/8 that constitutes critical downstream targets of BMP2 signaling [30, 31], we examined whether Panx3 is associated with BMP2-Smad signaling. To investigate the effect of endogenous Panx3 knockdown on the BMP signaling pathway, we examined phosphorylation levels of Smad1/5/8 in BMP2-treated mDP cells, which had been pretreated with siRNA or control siRNA. BMP2-induced phosphorylation of Smad1/5/8 was inhibited in *Panx3* siRNA treated cells (Fig 6). This inhibition was also observed in Panx3-specific hemichannel inhibitory peptide-treated cells (Fig 8C). These results indicate that Panx3 is involved in BMP-Smad signaling. Furthermore, we examined the effect of Panx3 on the activation of the MAPK superfamily, including JNK, p38, and ERK. However, the phosphorylation of JNK, p38, and ERK was not affected by the knockdown of endogenous Panx3 (data not shown).

### Panx3 promotes intracellular ATP release and AMPK activation

**Fig 7:**
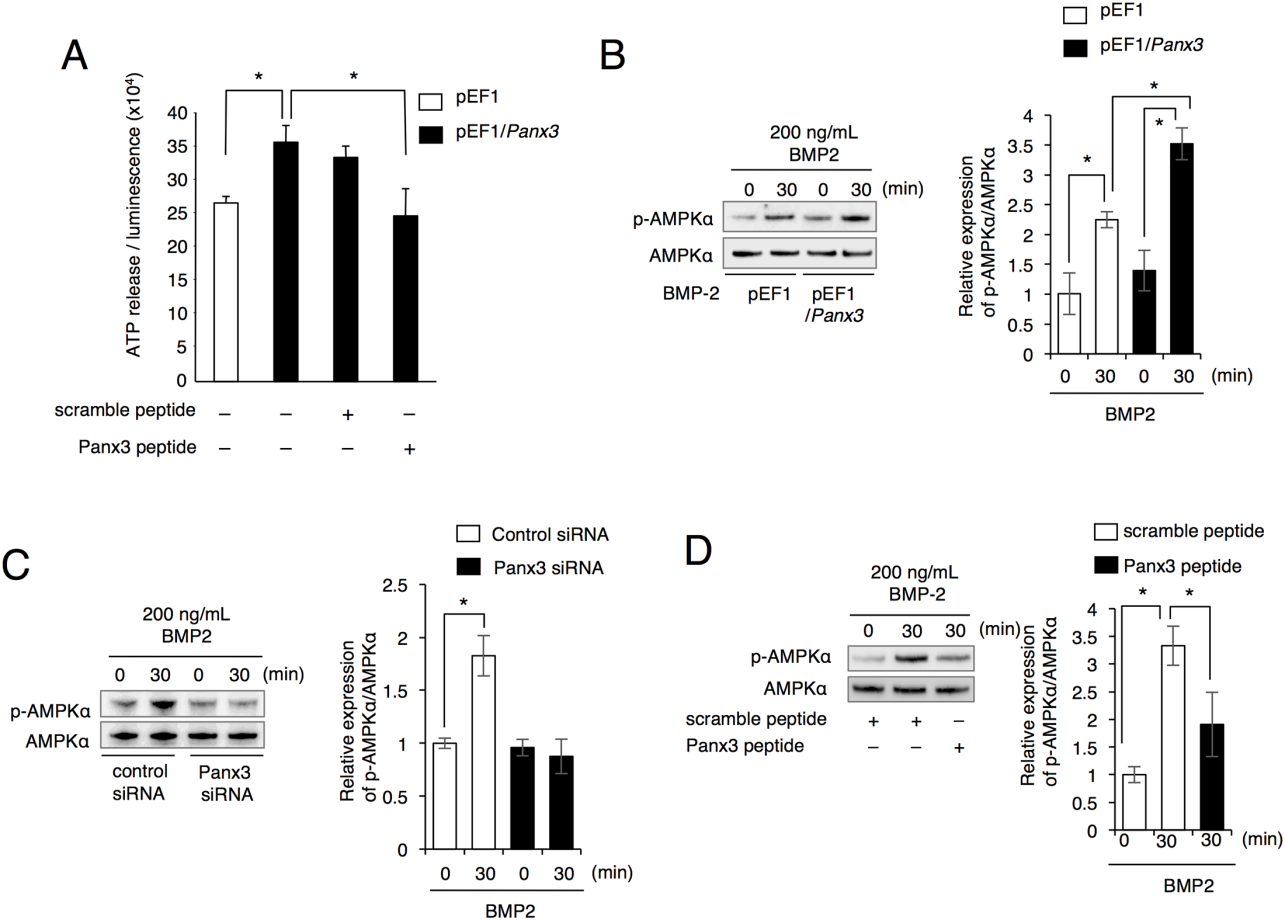
Increased ATP efflux and AMPK phosphorylation in Panx3-transfected mDP cells. (A) Cells were plated at ~50% confluency and ATP levels in the media were measured by luminometry. Statistical analysis was performed using analysis of variance (*P < 0.01). (B) Time course of AMPK phosphorylation in control pEF1 or pEF1/*Panx3*-transfected mDP cells following treatment with or without BMP2 was analyzed by western blotting using the anti-phospho-AMPK antibody. (C) Western blotting was performed using the anti-phospho-AMPK antibody for mDP cells transfected with either control siRNA or *Panx3* siRNA. (D) mDP cells were incubated with the Panx3 peptide or control peptide for 30 min, and then AMPK phosphorylation, induced by BMP2, was analyzed by western blotting. Data were pooled from three independent experiments with error bars designating standard deviation of the mean. Statistical analysis was performed using analysis of variance (*P < 0.01).

Since we previously reported that the Panx3 hemichannel releases intracellular ATP into the extracellular space in chondrocytes and osteoblasts [14, 15], we examined the efflux of ATP in mDP cells by luminometry. *Panx3*-transfected cells exhibited elevated ATP release compared to control vector-transfected cells. This Panx3 activity was inhibited by a Panx3-specific hemichannel blocking peptide but not by a control scrambled peptide (Fig 7A). These results suggest that Panx3 functions as an ATP release hemichannel in odontoblasts.

AMP-activated protein kinase (AMPK) is an energy-sensing kinase that plays a role in cellular energy homeostasis and is activated by intracellular ATP levels [32]. We tested whether the function of Panx3 as an ATP release hemichannel affects the activation of AMPK. Western blot analysis showed that BMP2 induced the phosphorylation of AMPK (Fig 7B). Furthermore, BMP2-mediated AMPK phosphorylation was further enhanced by Panx3 overexpression (Fig 7B). Moreover, both *Panx3* siRNA (Fig 7C) and Panx3-specific blocking peptide (Fig 7D) reduced BMP2-induced AMPK phosphorylation. These results indicate that Panx3 regulates AMPK activation.

### Relationship between BMP-Smad and Panx3-AMPK signaling in odontoblasts

**Fig 8:**
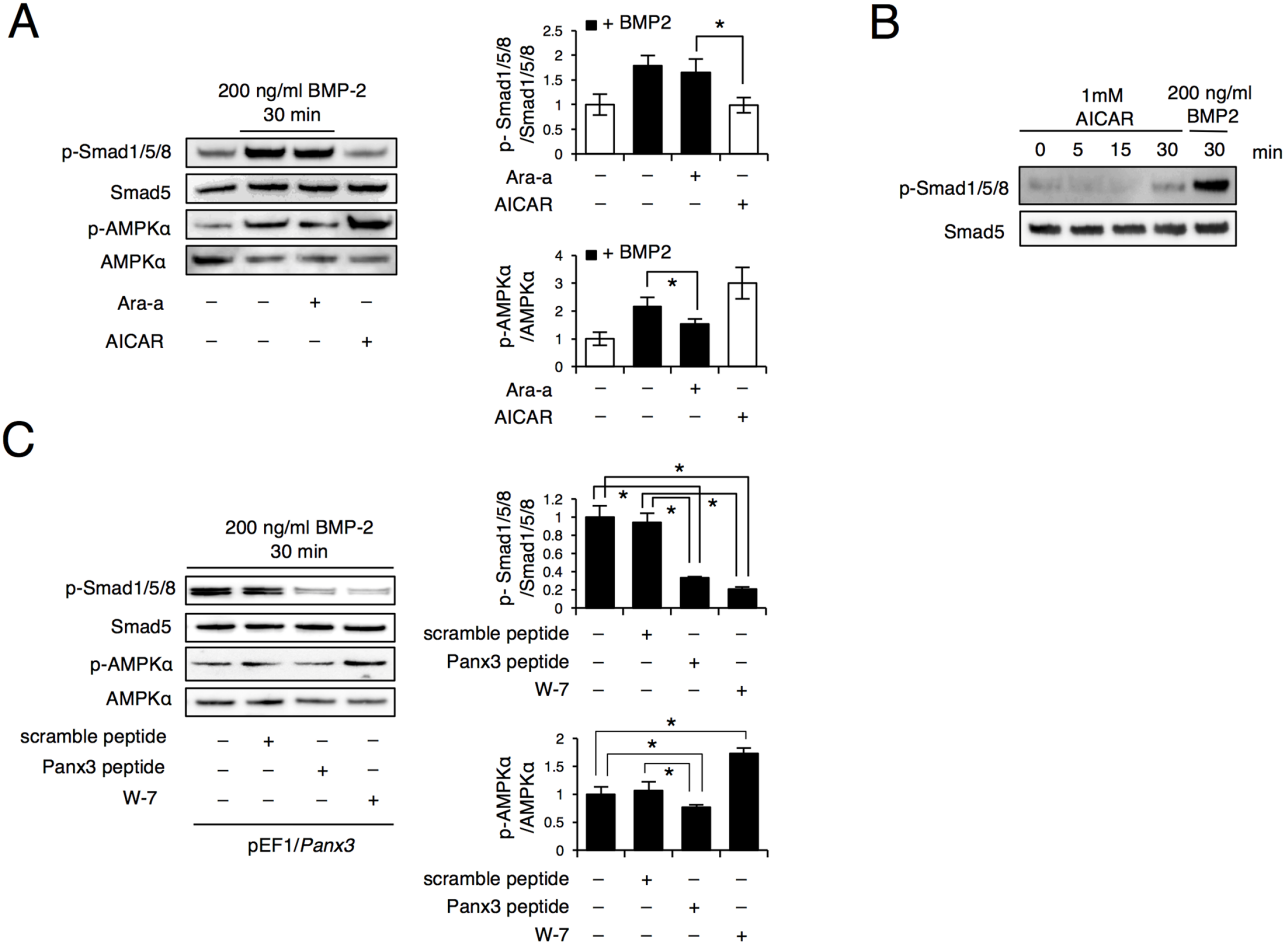
The role of Panx3 in AMPK and Smad signaling. (A) mDP cells were treated with 1 mM of Ara-a for 30 min before treatment with BMP2. 1 mM AICAR was tested Smad1/5/8 and AMPK phosphorylations by western blotting. Data were pooled from three independent experiments with error bars designating standard deviation of the mean. Statistical analysis was performed using analysis of variance (*P < 0.05). (B) The time course of Smad1/5/8 phosphorylation in mDP cells following treatment with AICAR was analyzed by western blotting using the anti-phospho-Smad1/5/8 antibody. (C) *Panx3*-transfected mDP cells were treated with either the Panx3 peptide or the calmodulin inhibitor W-7, and the phosphorylation of both Smad1/5/8 and AMPK was analyzed by western blotting. Data were pooled from three independent experiments with error bars designating standard deviation of the mean. Statistical analysis was performed using analysis of variance (*P < 0.05).

We next addressed whether BMP-Smad signaling is associated with the Panx3-AMPK pathway by examining the phosphorylation of Smad1/5/8 in the presence of the AMPK inhibitor Ara-a or the AMPK activator 5-Aminoimidazole-4-carboxamide-ribonucleoside (AICAR). BMP2 induced the phosphorylation of Smad1/5/8 and AMPK (Fig 8A). Ara-a treatment inhibited BMP2-induced AMPK phosphorylation but not Smad1/5/8 phosphorylation (Fig 8A). While AICAR induced AMPK phosphorylation, Smad1/5/8 phosphorylation was not induced (Fig 8A and 8B). These results indicate that BMP signaling influences AMPK signaling, but AMPK signaling does not affect BMP signaling. Furthermore, in Panx3 overexpression, when cells were treated with the Panx3 hemichannel blocking peptide, Smad1/5/8, but not AMPK phosphorylation were inhibited (Fig 8C). Panx3 functions as an endoplasmic reticulum (ER) Ca^2+^ channel [14, 15]. Akt activates the Panx3 ER Ca^2+^ channel that induces Ca^2+^ release from the ER into the cytosol, which subsequently activates calmodulin (CaM) signaling pathways including calmodulin-dependent protein kinase II (CaMKII)–Smad [15]. We investigated the effects of the CaM inhibitor W-7 (S3 Fig) on BMP2-induced Smad1/5/8 and AMPK phosphorylation in *Panx3*-transfected mDP cells. W-7 inhibited BMP2-induced Smad1/5/8 phosphorylation (Fig 8C). However, AMPK phosphorylation was not inhibited by W7 (Fig 8C). These results indicate that Panx3 regulates Smad signaling through the CaM signaling pathway in dental mesenchymal cells.

### Inhibition of cell proliferation by the AMPK activator AICAR

**Fig 9:**
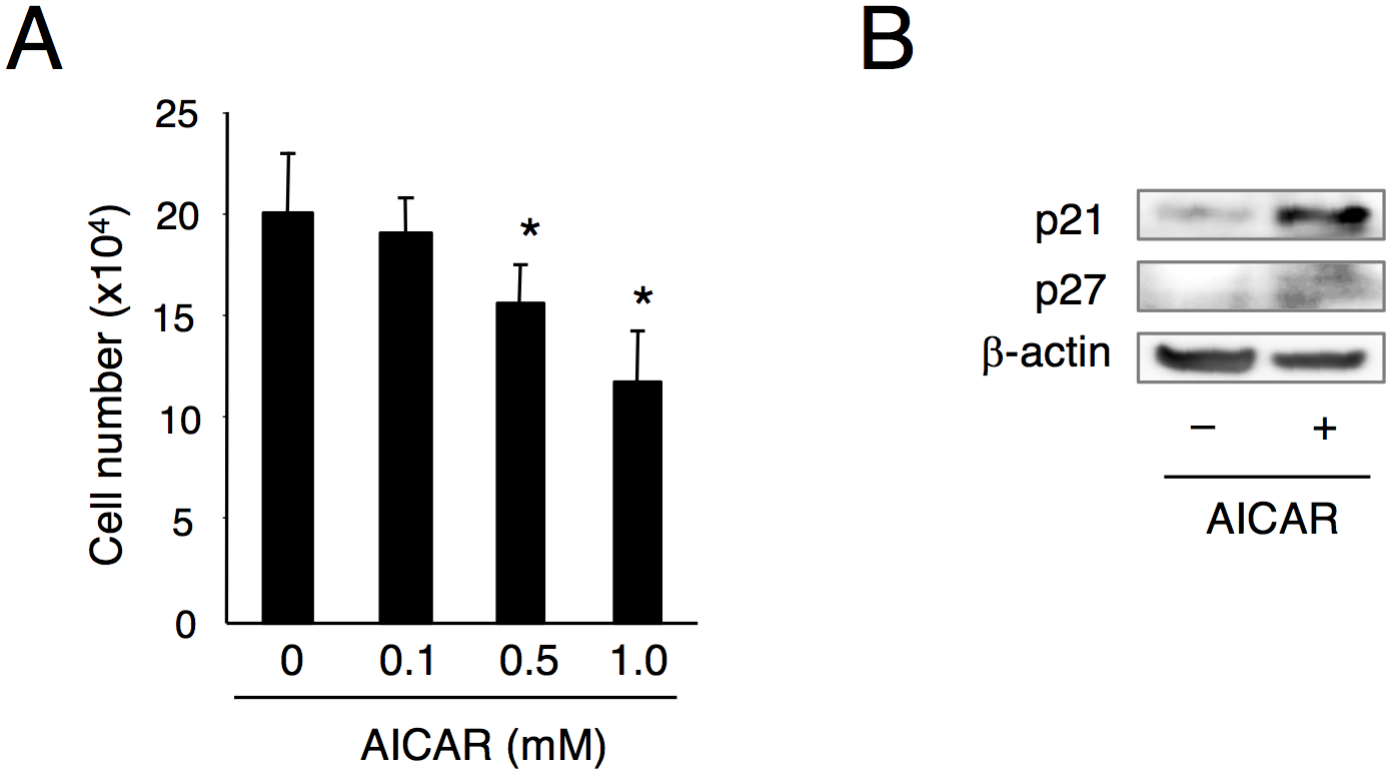
Inhibition of cell proliferation by AICAR. mDP cells were cultured in the presence of 0, 0.1, 0.5, or 1.0 mM 5-Aminoimidazole-4-carboxamide-ribonucleoside (AICAR), an AMPK activator, for three days and cell proliferation was measured using the cell counting kit-8. Data were pooled from three independent experiments with error bars designating standard deviation of the mean. Statistical analysis was performed using analysis of variance (*P < 0.01). (B) mDP cells were treated with 1.0 mM AICAR for three days and analyzed by western blotting using the antibodies of p21 and p27. β-actin was used as a control.

Finally, we examined whether AMPK signaling directly affects cell proliferation. The number of mDP cells decreased three days following the addition of AICAR in a dose dependent manner (Fig 9A). Moreover, we examined the effect of AICAR on the expression of p21 and p27. The expression of p21, but not p27, was induced by AICAR treatment (Fig 9B). These findings suggest that the AMPK pathway may be involved in the suppression of dental mesenchymal cell proliferation through p21 expression.

## Discussion

There are two dynamic and distinct cellular processes, growth arrest, and differentiation, during odontogenesis. The gradual differentiation of odontoblasts commences from the peripheral cell layer of the dental papilla [33]. Proliferating dental papilla cells eventually stop the cell cycle to differentiate into odontoblasts. Preodontoblasts are considered to be a regulator for growth arrest of proliferative dental papilla cells and for differentiation of odontoblasts. However, the precise function of preodontoblasts in tooth development is not clearly understood.

In this study, we found that pannexin 3 (Panx3) is specifically expressed in preodontoblasts. Differentiating odontoblasts secrete dentin matrix proteins including type I collagen and non-collagenous proteins such as Dspp. Dspp is an important molecule for dentin formation and is abundantly synthesized by differentiating odontoblasts. *Dspp*-null mice show defective and reduced dentin mineralization and pulp exposure similar to that of human dentinogenesis imperfecta III [34]. *Dspp* mutations demonstrate a direct correlation with human dentinogenesis imperfecta II and with dentin dysplasia II syndromes [35]. Thus, Dspp has been characterized as a unique marker of odontoblast differentiation. Here, we compared the expression of the *Panx3* mRNA and *Dspp* mRNA during tooth development. *In situ* hybridization revealed that *Panx3* mRNA expression completely differed from *Dspp* mRNA expression; *Panx3* mRNA-expressing cells did not express *Dspp* mRNA. Also we could not see any *Panx3*-positive signals in proliferating dental papilla cells. Thus, *Panx3* mRNA is specifically expressed in preodontoblasts.

Previously, we have demonstrated that Panx3 inhibits PTH-induced chondrogenic cell proliferation by regulating cAMP/PKA signaling [14]. We also demonstrated that Panx3 inhibits osteoprogenitor proliferation by inhibiting Wnt/β-catenin and PKA/CREB signaling and promotes cell cycle exit by increasing p21 activity (17). In this study, overexpression of *Panx3* significantly reduced mDP cell proliferation and induced p21 expression, whereas knockdown of endogenous *Panx3* reduced the expression of p21. Consistent with these findings, co-expression of p21 and Panx3 by preodontoblasts was observed by immunostaining. These results suggest that Panx3 may regulate cell proliferation via p21 expression in tooth development. Thus, Panx3 plays a central role in the regulation of proliferation of the progenitor cells in cartilage, bones, and teeth.

Panx3 functions as a hemichannel that releases intracellular ATP into the extracellular space of chondrocytes and osteoblasts [14–16]. We found that Panx3 also releases intracellular ATP as a hemichannel in mDP cells. The Panx3 hemichannel blocking peptide inhibited ATP release, indicating that the Panx3 hemichannel is involved in ATP release in preodontoblasts. Balancing intracellular ATP consumption and generation is one of the fundamental requirements of all cells. AMP-activated protein kinase (AMPK) serves as a highly conserved fuel sensor in all eukaryotic cells and is activated when intracellular ATP decreases. AMPK signaling plays an important role in many aspects of cellular functions, including cell proliferation [36–40]. In this study, we found that phosphorylation of AMPK was promoted by Panx3. In addition, we demonstrated that 5-Aminoimidazole-4-carboxamide-ribonucleoside (AICAR), an AMPK activator, significantly reduced mDP cell proliferation and induced p21 expression. These results suggest Panx3 mediates intracellular ATP release, which in turn leads to the activation of AMPK signaling, resulting in inhibition of cell proliferation by p21 expression in preodontoblasts. However, direct mechanism regulating p21 expression and cell cycle arrest via AMPK has been clearly defined. Further experiments were needed to resolve this hypothesis.

Panxs and connnexins share similar protein structures that consist of four hydrophobic transmembrane domains spaced by two extracellular loops, an intracellular loop, and intracellular amino (NH2) and carboxyl (COOH) temini. The gap junction protein features the protein structure to form a hexameric membrane pore complex and the hemichannel [41]. However, organ-, cell-, or time-specific expression of gap junction proteins are suggesting that each of gap junction proteins have specific roles and functions. In fact, connexin 43 (Cx43) plays important roles in osteoblast function and differentiation, but *Panx3* deficient mice show more severe skeletal phenotype than *Cx43* deficient mice [19]. This is due to the difference of channel properties between Panxs and connexins. Unlike connexins, the (endoplasmic reticulum) ER Ca^2+^ channel was found in only Panxs [13, 15, 17]. The ER plays a predominant role in Ca^2+^ storage and regulates intracellular Ca^2+^ levels in many cellular processes, including osteoblast differentiation. In osteoblasts, Panx3 functions as the ER Ca^2+^ channel that activates CaMKII-Smad signaling and *Osx* expression [15, 19]. Tooth development is mediated by various growth factors. Bone morphogenetic protein 2 (BMP2), one of the critical growth factors, is expressed in the primary enamel knot, an epithelial signaling center; BMP2 plays important roles in odontoblast differentiation involving *Dspp* expression [42]. We found that overexpression of *Panx3* promoted *Dspp* expression. In contrast, knockdown of *Panx3* reduced BMP2-induced Smad1/5/8 phosphorylation and *Dspp* expression. These results indicate that Panx3 is essential for odontoblast differentiation. Since the calmodulin inhibitor W-7 inhibited BMP2-induced Smad1/5/8 phosphorylation in this study, Panx3 may also function as the ER Ca^2+^ channel to increase intracellular Ca^2+^ levels for odontoblast differentiation.

**Fig 10:**
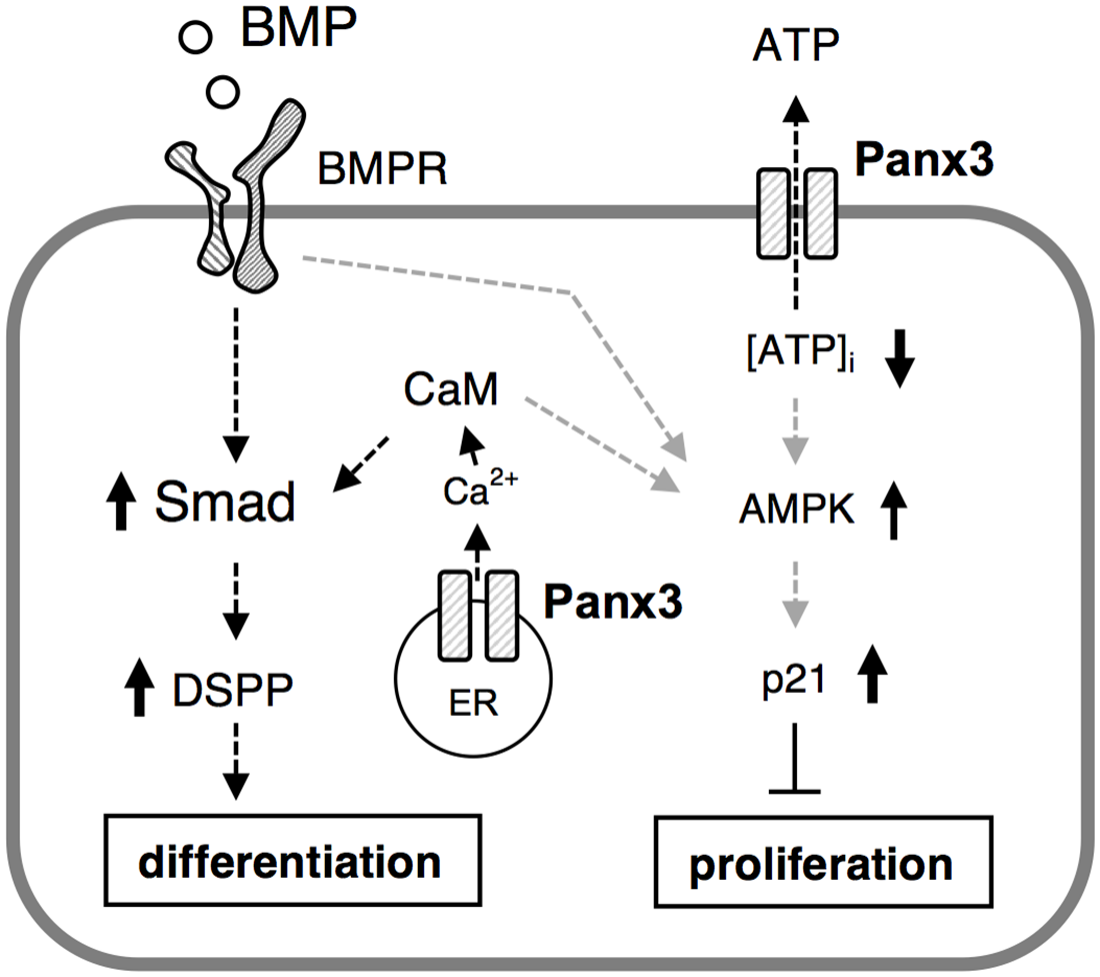
Role of Panx3 in odontoblast proliferation and differentiation. The Panx3 ER Ca^2+^ channel promotes BMP-Smad signaling for differentiation. Panx3 is expressed in preodontoblasts, and releases intracellular ATP to the extracellular space, which may result in an activation of AMPK and subsequent inhibition of cell proliferation.

In conclusion, this is the first study to demonstrate that Panx3 is specifically expressed in preodontoblast cells and regulates odontoblast cell proliferation and differentiation. The Panx3 hemichannel in preodontoblasts promotes ATP release into the extracellular space, which results in a reduction of intracellular ATP levels. It may be induced subsequent activation of AMPK-p21 signaling cascade to inhibit cell proliferation. The Panx3 hemichannel is also involved in calmodulin-Smad signaling for promotion of cell differentiation (Fig 10). Our results suggest that the restricted expression of Panx3 in preodontoblasts during odontoblast differentiation functions as a critical regulator for cell proliferation and differentiation. Further analysis of the regulatory role of Panx3 in preodontoblasts during odontogenesis may help elucidate the mechanisms underlying tooth development and provide innovative approaches to dentin regeneration.

## Supporting information

S1 Fig Expression of the Panx3 protein in mDP cells stimulated by BMP2. mDP cells were incubated in the presence of BMP2 for 48 h. The expression of Panx3 was analyzed by western blotting using the Panx3 antibody. β-actin was used as a control.
(TIFF)

S2 Fig Expression of Panx3 protein in mDP cells transfected with Panx3 siRNA. mDP cells transfected with either control siRNA or *Panx3* siRNA were cultured with 200 ng/mL BMP2 for 48 h. The expression of endogenous Panx3 was analyzed by western blotting using the Panx3 antibody. β-actin was used as a control. Statistical analysis was performed using analysis of variance (*P < 0.01).
(TIFF)

S3 Fig Inhibition of CaMKII phosphorylation by calmodulin inhibitor, W-7. SDP11 cells were pretreated with W-7 (10 μM) or DMSO as a control, and cultured with BMP2 (200 ng/mL) for 30 min. Cell extracts were analyzed by western blotting using anti-phospho-CaMKII antibody. β-actin was used as a control.
(TIFF)

## References

[1] Bennett MV, Verselis VK. Biophysics of gap junctions. Semin Cell Biol. 1992;3(1):29–47. Epub 1992/02/01. 1320429 10.1016/s1043-4682(10)80006-6

[2] Scemes E, Suadicani SO, Dahl G, Spray DC. Connexin and pannexin mediated cell-cell communication. Neuron Glia Biol. 2007;3(3):199–208. Epub 2008/07/19. 10.1017/S1740925X08000069 18634611 PMC2588549

[3] Bruzzone R, Hormuzdi SG, Barbe MT, Herb A, Monyer H. Pannexins, a family of gap junction proteins expressed in brain. Proc Natl Acad Sci U S A. 2003;100(23):13644–9. Epub 2003/11/05. 10.1073/pnas.2233464100 14597722 PMC263867

[4] Bruzzone R, White TW, Paul DL. Connections with connexins: the molecular basis of direct intercellular signaling. Eur J Biochem. 1996;238(1):1–27. Epub 1996/05/15. 8665925 10.1111/j.1432-1033.1996.0001q.x

[5] Lo CW, Cohen MF, Huang GY, Lazatin BO, Patel N, Sullivan R, et al Cx43 gap junction gene expression and gap junctional communication in mouse neural crest cells. Dev Genet. 1997;20(2):119–32. Epub 1997/01/01. 10.1002/(SICI)1520-6408(1997)20:2<119::AID-DVG5>3.0.CO;2-A 9144923

[6] Paznekas WA, Boyadjiev SA, Shapiro RE, Daniels O, Wollnik B, Keegan CE, et al Connexin 43 (GJA1) mutations cause the pleiotropic phenotype of oculodentodigital dysplasia. Am J Hum Genet. 2003;72(2):408–18. Epub 2002/11/29. 10.1086/346090 12457340 PMC379233

[7] Kelsell DP, Dunlop J, Stevens HP, Lench NJ, Liang JN, Parry G, et al Connexin 26 mutations in hereditary non-syndromic sensorineural deafness. Nature. 1997;387(6628):80–3. Epub 1997/05/01. 10.1038/387080a0 9139825

[8] Wu BL, Kenna M, Lip V, Irons M, Platt O. Use of a multiplex PCR/sequencing strategy to detect both connexin 30 (GJB6) 342 kb deletion and connexin 26 (GJB2) mutations in cases of childhood deafness. Am J Med Genet A. 2003;121A(2):102–8. Epub 2003/08/12. 10.1002/ajmg.a.20210 12910486

[9] Reaume AG, de Sousa PA, Kulkarni S, Langille BL, Zhu D, Davies TC, et al Cardiac malformation in neonatal mice lacking connexin43. Science. 1995;267(5205):1831–4. 7892609 10.1126/science.7892609

[10] Buhl DL, Harris KD, Hormuzdi SG, Monyer H, Buzsaki G. Selective impairment of hippocampal gamma oscillations in connexin-36 knock-out mouse in vivo. J Neurosci. 2003;23(3):1013–8. Epub 2003/02/08. 12574431 10.1523/JNEUROSCI.23-03-01013.2003 PMC6741916

[11] Baranova A, Ivanov D, Petrash N, Pestova A, Skoblov M, Kelmanson I, et al The mammalian pannexin family is homologous to the invertebrate innexin gap junction proteins. Genomics. 2004;83(4):706–16. Epub 2004/03/19. 10.1016/j.ygeno.2003.09.025 15028292

[12] Bao L, Locovei S, Dahl G. Pannexin membrane channels are mechanosensitive conduits for ATP. FEBS Lett. 2004;572(1–3):65–8. Epub 2004/08/12. 10.1016/j.febslet.2004.07.009 15304325

[13] Vanden Abeele F, Bidaux G, Gordienko D, Beck B, Panchin YV, Baranova AV, et al Functional implications of calcium permeability of the channel formed by pannexin 1. J Cell Biol. 2006;174(4):535–46. 10.1083/jcb.200601115 16908669 PMC2064259

[14] Iwamoto T, Nakamura T, Doyle A, Ishikawa M, de Vega S, Fukumoto S, et al Pannexin 3 regulates intracellular ATP/cAMP levels and promotes chondrocyte differentiation. J Biol Chem. 2010;285(24):18948–58. Epub 2010/04/21. 10.1074/jbc.M110.127027 20404334 PMC2881817

[15] Ishikawa M, Iwamoto T, Nakamura T, Doyle A, Fukumoto S, Yamada Y. Pannexin 3 functions as an ER Ca(2+) channel, hemichannel, and gap junction to promote osteoblast differentiation. J Cell Biol. 2011;193(7):1257–74. Epub 2011/06/22. 10.1083/jcb.201101050 21690309 PMC3216329

[16] Ishikawa M, Iwamoto T, Fukumoto S, Yamada Y. Pannexin 3 inhibits proliferation of osteoprogenitor cells by regulating Wnt and p21 signaling. J Biol Chem. 2014;289(5):2839–51. 10.1074/jbc.M113.523241 24338011 PMC3908416

[17] Bond SR, Lau A, Penuela S, Sampaio AV, Underhill TM, Laird DW, et al Pannexin 3 is a novel target for Runx2, expressed by osteoblasts and mature growth plate chondrocytes. J Bone Miner Res. 2011;26(12):2911–22. Epub 2011/09/15. 10.1002/jbmr.509 21915903

[18] Oh SK, Shin JO, Baek JI, Lee J, Bae JW, Ankamerddy H, et al Pannexin 3 is required for normal progression of skeletal development in vertebrates. FASEB J. 2015;29(11):4473–84. 10.1096/fj.15-273722 26183770

[19] Ishikawa M, Williams GL, Ikeuchi T, Sakai K, Fukumoto S, Yamada Y. Pannexin 3 and connexin 43 modulate skeletal development via distinct functions and expression patterns. J Cell Sci. 2016. 10.1242/jcs.176883 PMC4813316 26759176

[20] Sasaki T, Garant PR. Structure and organization of odontoblasts. Anat Rec. 1996;245(2):235–49. 10.1002/(SICI)1097-0185(199606)245:2<235::AID-AR10>3.0.CO;2-Q 8769666

[21] Ushiyama J. Gap junctions between odontoblasts revealed by transjunctional flux of fluorescent tracers. Cell Tissue Res. 1989;258(3):611–6. 2482135 10.1007/BF00218874

[22] Fried K, Mitsiadis TA, Guerrier A, Haegerstrand A, Meister B. Combinatorial expression patterns of the connexins 26, 32, and 43 during development, homeostasis, and regeneration of rat teeth. Int J Dev Biol. 1996;40(5):985–95. Epub 1996/10/01. 8946246

[23] Joao SM, Arana-Chavez VE. Expression of connexin 43 and ZO-1 in differentiating ameloblasts and odontoblasts from rat molar tooth germs. Histochem Cell Biol. 2003;119(1):21–6. 12548402 10.1007/s00418-002-0482-3

[24] de Vega S, Iwamoto T, Nakamura T, Hozumi K, McKnight DA, Fisher LW, et al TM14 is a new member of the fibulin family (fibulin-7) that interacts with extracellular matrix molecules and is active for cell binding. The Journal of biological chemistry. 2007;282(42):30878–88. 10.1074/jbc.M705847200 17699513

[25] Yuasa K, Fukumoto S, Kamasaki Y, Yamada A, Fukumoto E, Kanaoka K, et al Laminin alpha2 is essential for odontoblast differentiation regulating dentin sialoprotein expression. The Journal of biological chemistry. 2004;279(11):10286–92. 10.1074/jbc.M310013200 14681233

[26] Nakamura T, Unda F, de-Vega S, Vilaxa A, Fukumoto S, Yamada KM, et al The Kruppel-like factor epiprofin is expressed by epithelium of developing teeth, hair follicles, and limb buds and promotes cell proliferation. The Journal of biological chemistry. 2004;279(1):626–34. 10.1074/jbc.M307502200 14551215

[27] Lee DS, Park JT, Kim HM, Ko JS, Son HH, Gronostajski RM, et al Nuclear factor I-C is essential for odontogenic cell proliferation and odontoblast differentiation during tooth root development. J Biol Chem. 2009;284(25):17293–303. Epub 2009/04/24. 10.1074/jbc.M109.009084 19386589 PMC2719365

[28] Arakaki M, Ishikawa M, Nakamura T, Iwamoto T, Yamada A, Fukumoto E, et al Role of epithelial-stem cell interactions during dental cell differentiation. J Biol Chem. 2012. Epub 2012/02/03. 10.1074/jbc.M111.285874 PMC3323010 22298769

[29] Akazawa Y, Hasegawa T, Yoshimura Y, Chosa N, Asakawa T, Ueda K, et al Recruitment of mesenchymal stem cells by stromal cell-derived factor 1alpha in pulp cells from deciduous teeth. Int J Mol Med. 2015;36(2):442–8. 10.3892/ijmm.2015.2247 26082290

[30] Qin W, Yang F, Deng R, Li D, Song Z, Tian Y, et al Smad 1/5 is involved in bone morphogenetic protein-2-induced odontoblastic differentiation in human dental pulp cells. J Endod. 2012;38(1):66–71. Epub 2011/12/14. 10.1016/j.joen.2011.09.025 22152623

[31] Ogasawara T, Kawaguchi H, Jinno S, Hoshi K, Itaka K, Takato T, et al Bone morphogenetic protein 2-induced osteoblast differentiation requires Smad-mediated down-regulation of Cdk6. Mol Cell Biol. 2004;24(15):6560–8. Epub 2004/07/16. 10.1128/MCB.24.15.6560-6568.2004 15254224 PMC444857

[32] Mihaylova MM, Shaw RJ. The AMPK signalling pathway coordinates cell growth, autophagy and metabolism. Nat Cell Biol. 2011;13(9):1016–23. Epub 2011/09/06. 10.1038/ncb2329 21892142 PMC3249400

[33] Fukumoto E, Sakai H, Fukumoto S, Yagi T, Takagi O, Kato Y. Cadherin-related neuronal receptors in incisor development. Journal of dental research. 2003;82(1):17–22. 12508039 10.1177/154405910308200105

[34] Sreenath T, Thyagarajan T, Hall B, Longenecker G, D'Souza R, Hong S, et al Dentin sialophosphoprotein knockout mouse teeth display widened predentin zone and develop defective dentin mineralization similar to human dentinogenesis imperfecta type III. J Biol Chem. 2003;278(27):24874–80. Epub 2003/05/02. 10.1074/jbc.M303908200 12721295

[35] McKnight DA, Simmer JP, Hart PS, Hart TC, Fisher LW. Overlapping DSPP mutations cause dentin dysplasia and dentinogenesis imperfecta. J Dent Res. 2008;87(12):1108–11. Epub 2008/11/26. 10.1177/154405910808701217 19029076 PMC2596760

[36] Gwinn DM, Shackelford DB, Egan DF, Mihaylova MM, Mery A, Vasquez DS, et al AMPK phosphorylation of raptor mediates a metabolic checkpoint. Mol Cell. 2008;30(2):214–26. Epub 2008/04/29. 10.1016/j.molcel.2008.03.003 18439900 PMC2674027

[37] Kalender A, Selvaraj A, Kim SY, Gulati P, Brule S, Viollet B, et al Metformin, independent of AMPK, inhibits mTORC1 in a rag GTPase-dependent manner. Cell Metab. 2010;11(5):390–401. Epub 2010/05/07. 10.1016/j.cmet.2010.03.014 20444419 PMC3081779

[38] Jones RG, Plas DR, Kubek S, Buzzai M, Mu J, Xu Y, et al AMP-activated protein kinase induces a p53-dependent metabolic checkpoint. Mol Cell. 2005;18(3):283–93. Epub 2005/05/04. 10.1016/j.molcel.2005.03.027 15866171

[39] Liang J, Shao SH, Xu ZX, Hennessy B, Ding Z, Larrea M, et al The energy sensing LKB1-AMPK pathway regulates p27(kip1) phosphorylation mediating the decision to enter autophagy or apoptosis. Nat Cell Biol. 2007;9(2):218–24. Epub 2007/01/24. 10.1038/ncb1537 17237771

[40] Bjorklund MA, Vaahtomeri K, Peltonen K, Viollet B, Makela TP, Band AM, et al Non-CDK-bound p27 (p27(NCDK)) is a marker for cell stress and is regulated through the Akt/PKB and AMPK-kinase pathways. Exp Cell Res. 2010;316(5):762–74. Epub 2009/12/29.] 10.1016/j.yexcr.2009.12.014 20036235

[41] Unger VM, Kumar NM, Gilula NB, Yeager M. Three-dimensional structure of a recombinant gap junction membrane channel. Science. 1999;283(5405):1176–80. 10024245 10.1126/science.283.5405.1176

[42] Thesleff I. Epithelial-mesenchymal signalling regulating tooth morphogenesis. J Cell Sci. 2003;116(Pt 9):1647–8. Epub 2003/04/01. 12665545 10.1242/jcs.00410

