# Supplementary figures and images for: Pannexin 3 regulates proliferation and differentiation of odontoblasts via its hemichannel activities
Source: PLoS One. 2017 May 11;12(5):e0177557. doi: 10.1371/journal.pone.0177557 (PMC5426780; doi:10.1371/journal.pone.0177557)

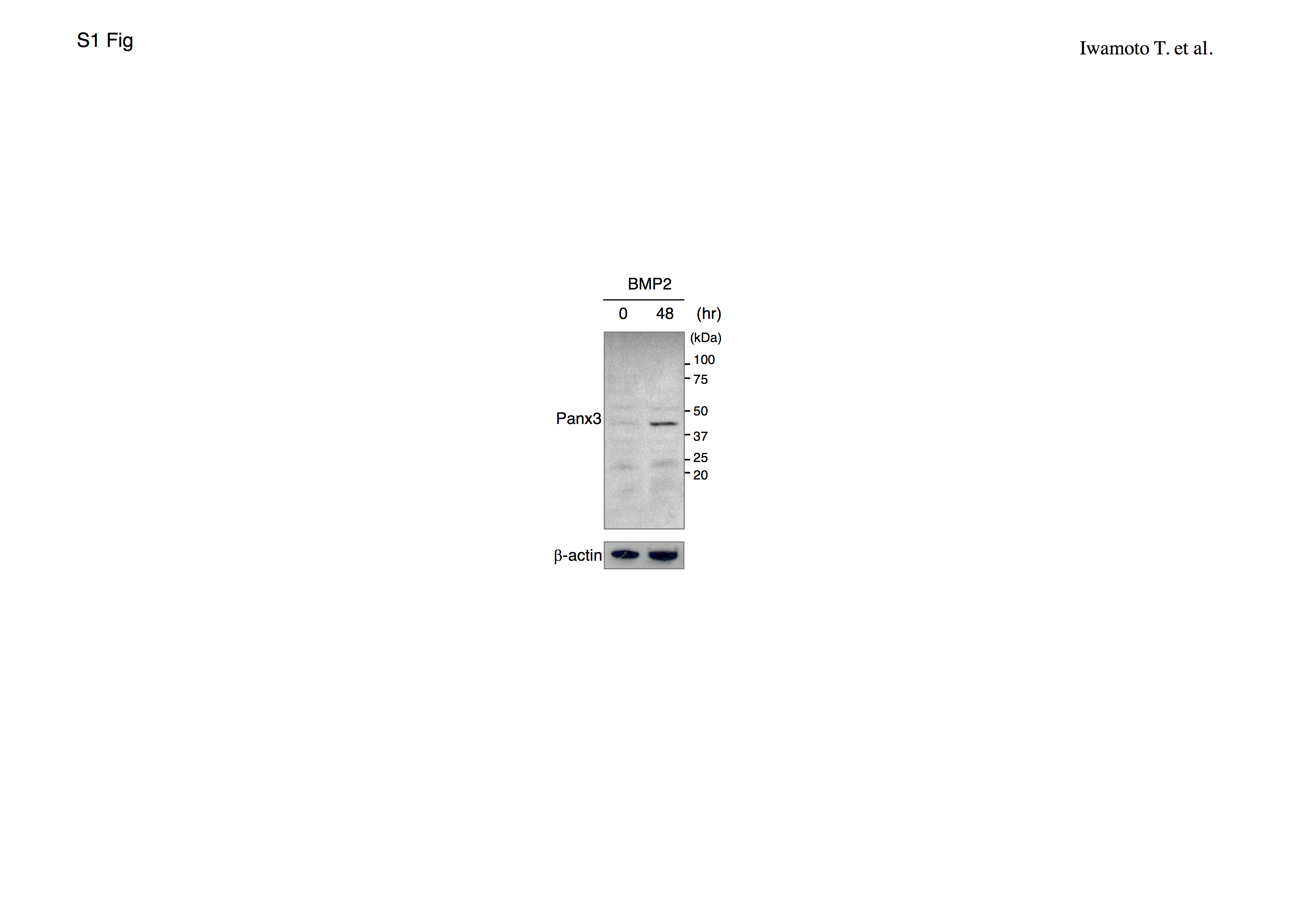

Supplement: S1 Fig — mDP cells were incubated in the presence of BMP2 for 48 h. The expression of Panx3 was analyzed by western blotting using the Panx3 antibody. β-actin was used as a control. (TIFF) [file pone.0177557.s001.tiff]

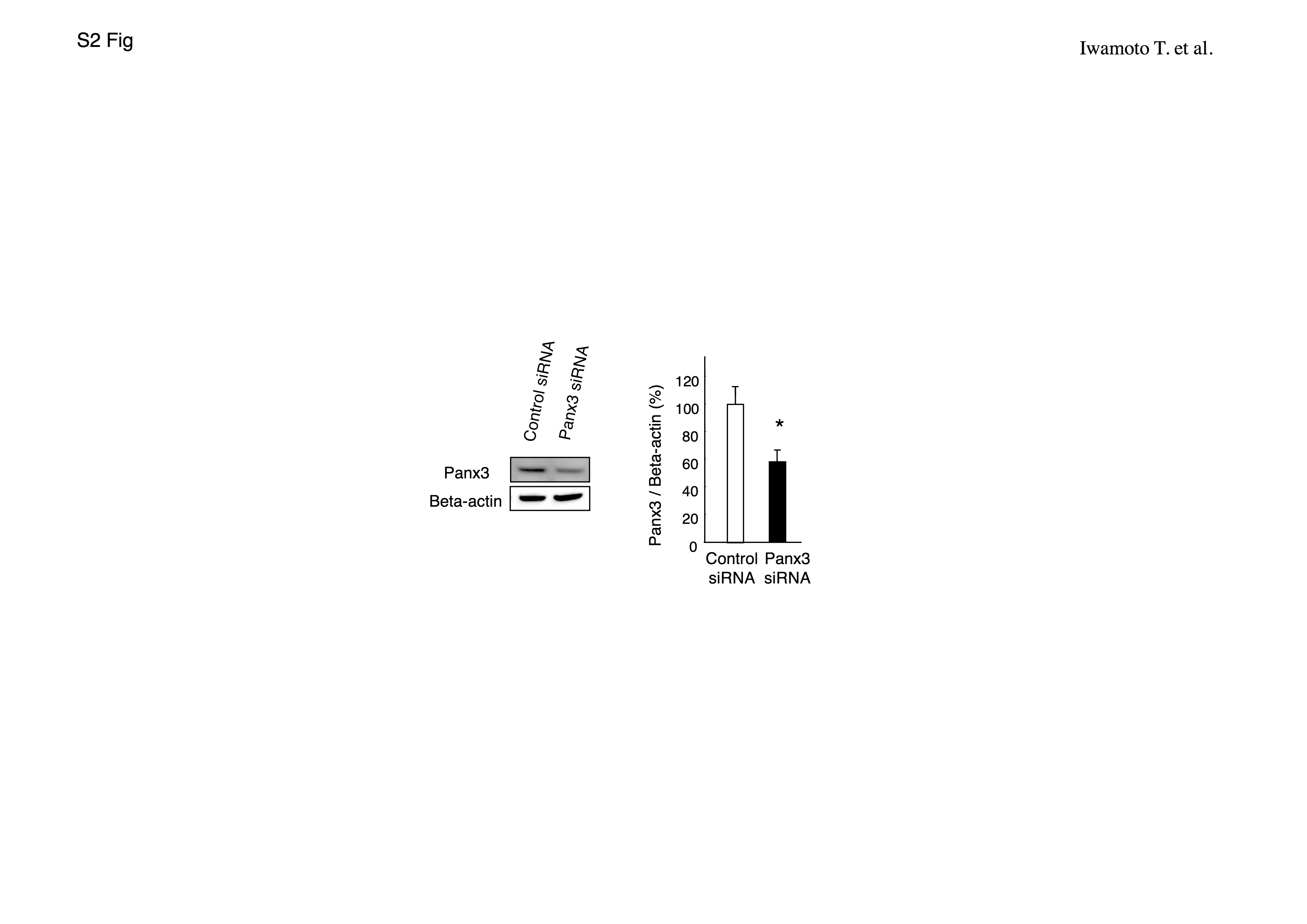

Supplement: S2 Fig — mDP cells transfected with either control siRNA or Panx3 siRNA were cultured with 200 ng/mL BMP2 for 48 h. The expression of endogenous Panx3 was analyzed by western blotting using the Panx3 antibody. β-actin was used as a control. Statistical analysis was performed using analysis of variance (*P < 0.01). (TIFF) [file pone.0177557.s002.tiff]

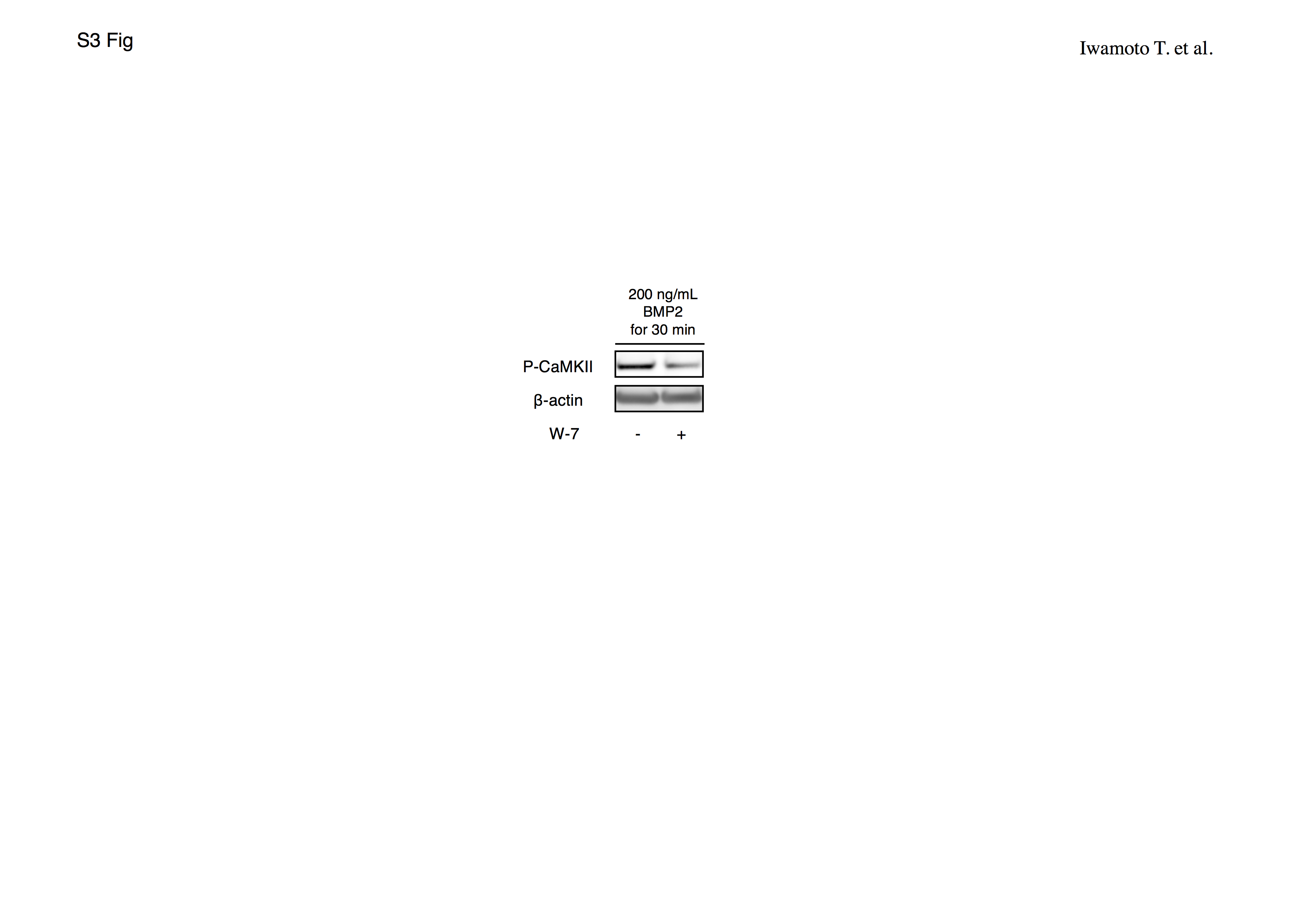

Supplement: S3 Fig — SDP11 cells were pretreated with W-7 (10 μM) or DMSO as a control, and cultured with BMP2 (200 ng/mL) for 30 min. Cell extracts were analyzed by western blotting using anti-phospho-CaMKII antibody. β-actin was used as a control. (TIFF) [file pone.0177557.s003.tiff]
